# Hypercontractile phenotype at rest in chronic coronary syndromes predicts impaired functional reserve and increased mortality

**DOI:** 10.1093/eschf/xvag151

**Published:** 2026-06-06

**Authors:** Quirino Ciampi, Yi Wang, Lauro Cortigiani, Angela Zagatina, Ratnasari Padang, Garvan C Kane, Hector R Villarraga, Adelaide M Arruda-Olson, Elena Kalinina, Jorge Lowenstein, Rosina Arbucci, Diego M Lowenstein Haber, Sofia Marconi, Jelena Celutkiene, Alla Boshchenko, Tamara Ryabova, Hugo Rodriguez-Zanella, Francesca Bursi, Karina Wierzbowska-Drabik, Jaroslaw D Kasprzak, Elisa Merli, Ana Djordjevic-Dikic, Miguel Amor, Maciej Haberka, Ariel Saad, Ayten Safarova, Tatiana Timofeeva, Jesus Peteiro Vazquez, Fausto Rigo, Aleksandra Nikolic, Miodrag Ostojic, Nicola Gaibazzi, Tamara Kovacevic-Preradovic, Emma Cerracchio, Giovanni Luigi Tripepi, Lixue Yin, Bruno Villari, Mauro Pepi, Scipione Carerj, Patricia A Pellikka, Eugenio Picano

**Affiliations:** Division of Cardiology, Fatebenefratelli Hospital, Viale Principe di Napoli, 12, Benevento I-82100, Italy; Department of Cardiovascular Ultrasound and Non-Invasive Cardiology, Sichuan Provincial People's Hospital, Chengdu, China; Cardiology Department, and Aesculapius Medical Center San Luca Hospital, Lucca, Italy; Cardiosurgery Department, St.Petersburg State University, Saint Petersburg, Russian Federation; Department of Cardiovascular Medicine, Mayo Clinic, Rochester, MN, USA; Department of Cardiovascular Medicine, Mayo Clinic, Rochester, MN, USA; Department of Cardiovascular Medicine, Mayo Clinic, Rochester, MN, USA; Department of Cardiovascular Medicine, Mayo Clinic, Rochester, MN, USA; Cardiosurgery Department, St.Petersburg State University, Saint Petersburg, Russian Federation; Cardiodiagnosticos, Investigaciones Medicas, Buenos Aires, Argentina; Cardiodiagnosticos, Investigaciones Medicas, Buenos Aires, Argentina; Cardiodiagnosticos, Investigaciones Medicas, Buenos Aires, Argentina; Cardiodiagnosticos, Investigaciones Medicas, Buenos Aires, Argentina; Centre of Cardiology and Angiology, Clinic of Cardiac and Vascular Diseases, Faculty of Medicine, Institute of Clinical Medicine, Vilnius University, State Research Institute Center for Innovative Medicine, Vilnius, Lithuania; Cardiology Research Institute, Tomsk National Research Medical Centre of the Russian Academy of Sciences, Tomsk, Russian Federation; Cardiology Research Institute, Tomsk National Research Medical Centre of the Russian Academy of Sciences, Tomsk, Russian Federation; Cardiology Department, Instituto Nacional de Cardiologia Ignacio Chavez, Mexico City, Mexico; Department of Health Sciences, Cardiology Division, University of Milan, ASST Santi Paolo e Carlo, Milan, Italy; Department of Internal Diseases and Clinical Pharmacology, Medical University, Lodz, Poland; I Department of Cardiology, Bieganski Hospital, Medical University, Lodz, Poland; Cardiology Unit, Ospedale per gli Infermi, Faenza, and Department of Cardiovascular Disease-AUSL Romagna, Division of Cardiology, Ospedale S. Maria delle Croci, Ravenna, Italy; Cardiology Clinic, University Center Serbia, Medical School, University Clinical Center Serbia, University of Belgrade, Belgrade, Serbia; Hospital Echocardiography Laboratory, Ramos Mejia Hospital, Buenos Aires, Argentina; Cardiology Department, University of Silesia, Katowice, Poland; División de Cardiología, Hospital de Clínicas José de San Martín, Buenos Aires, Argentina; Department of Internal Medicine with a Course in Cardiology and Functional Diagnostics at the Medical Institute of the Peoples’ Friendship University of Russia, Moscow, Russia; Department of Internal Medicine with a Course in Cardiology and Functional Diagnostics at the Medical Institute of the Peoples’ Friendship University of Russia, Moscow, Russia; Cardiology Department, CHUAC-Complexo Hospitalario Universitario A Coruna-University of A Coruna, La Coruna, Spain; Cardiology Department, Vlla Salus Foundation/IRCCS San Camillo Hospital, Venice Venezia, Italy; Department of Noninvasive Cardiology, Institute for Cardiovascular Diseases Dedinje, School of Medicine, Belgrade, Serbia; Department of Noninvasive Cardiology, Institute for Cardiovascular Diseases Dedinje, School of Medicine, Belgrade, Serbia; Cardiology Department, University of Parma, Parma, Italy; Clinic of Cardiovascular Diseases, University of Banja Luka University Clinical Centre of the Republic of Srpska, Banja Luka, Republic of Srpska; Division of Cardiology, Fatebenefratelli Hospital, Viale Principe di Napoli, 12, Benevento I-82100, Italy; CNR, Institute of Clinical Physiology, Reggio Calabria, Italy; Department of Cardiovascular Ultrasound and Non-Invasive Cardiology, Sichuan Provincial People's Hospital, Chengdu, China; Division of Cardiology, Fatebenefratelli Hospital, Viale Principe di Napoli, 12, Benevento I-82100, Italy; Centro Cardiologico Monzino, IRCCS, Milano, Italy; Cardiology Division, University Hospital Policlinico G. Martino, University of Messina, Messina, Italy; Department of Cardiovascular Medicine, Mayo Clinic, Rochester, MN, USA; Cardiology Clinic, University Center Serbia, Medical School, University Clinical Center Serbia, University of Belgrade, Belgrade, Serbia

**Keywords:** Echocardiography, Elastance, Force, Outcome, Survival

## Abstract

**Introduction:**

Transthoracic echocardiography (TTE) identifies a hypercontractile phenotype (HP) in chronic coronary syndromes (CCS), characterized by elevated resting left ventricular (LV) elastance (force = systolic blood pressure/end-systolic volume). To evaluate the prognostic significance and functional correlates of HP.

**Methods:**

In a prospective multicentre study, 10 677 patients with CCS underwent resting TTE to assess LV ejection fraction (EF), stroke volume, and force by quantitative volumetric echocardiography. All patients were followed for the endpoint of all-cause mortality. In a subset of 5834 patients, stress echocardiography (exercise or dobutamine) was performed for LV contractile reserve and heart rate reserve.

**Results:**

Patients were stratified into Force quintiles (Q1–Q5). Patients with hypercontractile phenotype exhibited lower stroke volume at rest (Q5 = 34.8 ± 12.3 vs Q1–Q4 = 57.4 ± 19.1 mL; *P* < .01) and higher EF at rest (Q5 = 64.8 ± 6.9% vs Q1–Q4 = 58.1 ± 8.7%, *P* < .01). During a median follow-up of 24 months (interquartile range = 12–40 months), 509 deaths occurred. The exposure-adjusted death rate was lowest in Q3 (3.53–4.51 mmHg/mL; 1.03 per 100 person/years) and higher in Q1 (≤2.62 mmHg/mL, 2.88), Q2 (2.63–3.52 mmHg/mL, 1.86), Q4 (4.52–6.11 mmHg/mL, 1.56), and Q5 (HP, >6.11 mmHg/mL; 1.88; *P* < .0001 vs Q1 and Q3). Multivariable analysis identified HP (Q5; HR 1.531 vs Q3, 95% CI 1.116–2.099; *P* = .006) and EF (HR 0.963, 95% CI 0.953–0.972; *P* < .0001) as independent predictors of death. During exercise or dobutamine stress, HP showed reduced LV contractile reserve (ΔEF: Q5 = 4.3 ± 9.4% vs Q1–Q4 = 7.0 ± 9.3%; *P* < .001) and blunted heart rate reserve (Q5 = 1.77 ± 0.33 vs Q1–Q4 = 1.85 ± 0.39; *P* < .01). All patients with force-based LV contractile reserve >4.1 (present in 185, 3.2% of the population) survived.

**Conclusion:**

Patients with CCS with HP assessed by resting TTE demonstrate higher mortality and multilayered functional impairment, including reduced LV contractile and chronotropic reserves. Hypercontractile phenotype improved the prediction of mortality by EF. A ‘stronger’ heart is, in fact, functionally and prognostically weaker.

## Graphical Abstract

**Graphical Abstract:**
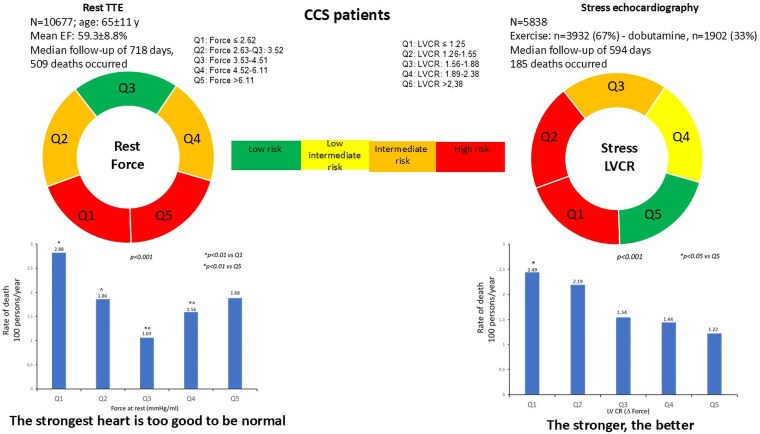
On the left, the data of rest TTE: a heart with HP is too good to be normal. On the right, the data with SE: the stronger the heart (with greater LVCR), the better the outcome.

## Introduction

The left ventricular (LV) hypercontractile phenotype (HP) arises from genetic sarcomeric mutations, altered Ca^2+^ handling, and hyperadrenergic states, leading to enhanced systolic function but impaired diastolic filling.^1^ Hypercontractile phenotype becomes pathological in chronic coronary syndromes (CCS), hypertrophic cardiomyopathy, hypertension, or heart failure with preserved ejection fraction (EF), increasing the risk of heart failure and arrhythmias.^2^

Transthoracic echocardiography (TTE) is helpful to identify HP in several possible ways as increased (>70%) EF, global longitudinal strain absolute values >25%, or with the relatively load-independent index of high resting left ventricular (LV) end-systolic elastance (Ees, also called force), measured as the ratio of systolic blood pressure (SBP)/end-systolic volume (ESV).^3^ HP identified with resting TTE is associated with a small heart with reduced end-diastolic volume, reduced stroke volume (SV) despite high EF, and higher arterial elastance in patients with CCS,^4^ heart failure with preserved EF,^5^ and in hypertrophic cardiomyopathy.^6^

While LV elastance is a true load-independent index of LV function, it requires the assessment of the slope (and therefore at least 2 points) to measure pressure and volume under different loading conditions. Left ventricular force is a simplified, load-dependent estimate of LV function that changes with afterload and is more prognostically accurate than EF.

The study hypothesis was that the resting HP identified as the highest quintile (Q) of force in the distribution of the enrolled population was associated with increased functional vulnerability evaluated with stress echocardiography (SE) and worse survival in all-comers with CCS. To test this hypothesis, data from the international, multicentre, prospective SE 2030 study were interrogated.^7^

## Methods

The study protocol was reviewed and approved by the institutional ethics committees, as a part of the more comprehensive SE 2020 study (148-Comitato Etico Lazio-1, 16 July 2016; ClinicalTrials.gov Identifier NCT 030.49995) and SE 2030 study (291/294/295 Comitato Etico Lazio-1, 8 March 2021; ClinicalTrials.gov Identifier NCT NCT050.81115). No support from the industry was received. The study was officially endorsed by the Italian Society of Echocardiography and Cardiovascular Imaging (SIECVI), and all data were stored in a dedicated data bank property of SIECVI. Written informed consent was obtained from all patients. The data that support the findings of this study are available from the corresponding author upon reasonable request.

### Study population

**Figure 1:**
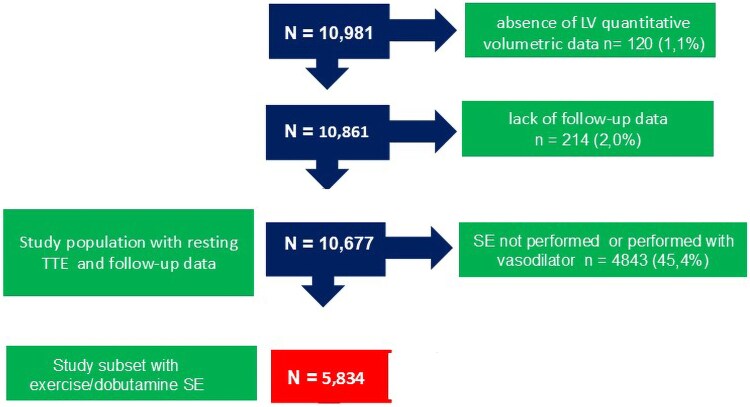
Flowchart of this study. CAD, coronary artery disease; EDV, end-diastolic volume; ESV, end-systolic volume; SBP, systolic blood pressure; SE, stress echocardiography; WMSI, wall motion score index

In this retrospective analysis of prospectively acquired data, we initially screened 10 981 CCS, patients were recruited from July 2016 to May 2025 by 32 certified laboratories across 12 countries (Argentina, Bosnia-Herzegovina Republic of Srpska, Bulgaria, Hungary, Italy, Lithuania, Mexico, Poland, the Russian Federation, Serbia, Spain, USA). The initial inclusion criteria were (1) age >18 years; (2) referral for known or suspected CCS; (3) no severe valvular or congenital heart disease, or presence of prognosis-limiting comorbidities, such as advanced cancer, reducing life expectancy to <1 year; (4) TTE of acceptable quality at rest; and (5) willingness to give written informed consent allowing scientific utilization of observational data, respectful of privacy rights. Exclusion criteria were the following: (1) absence of LV quantitative volumetric data (*n* = 120, 1.1%) or (2) lack of follow-up data (*n* = 214, 2.0%). The final study population consisted of 10 677 patients, all of whom were studied with rest TTE and had available follow-up information (*Figure 1*). In a subset of 5834 patients, SE data (exercise or dobutamine) were available.

All patients underwent comprehensive TTE at rest following standard recommendations, including measurement of LV end-diastolic volume and ESV with biplane Simpson’s method^8^ or apical single plane area-length method,^9^ and EF and SV as previously described.^4,5^ The lung ultrasound acquisition was performed at rest with the 4-site simplified scan at the third intercostal space on the anterior and lateral hemithoraces, using the same probe employed for the cardiac scan. After scanning the four chest sites, the cumulative B-line score was obtained by summing the number of detected B-lines at each site. B-lines were considered present if at least two B-lines could be detected.^5,6^ Commercially available ultrasound systems were used.

Stress echocardiography was performed in 3932 patients (67.4%) using exercise and in 1902 patients (32.6%) using dobutamine, adhering to the protocols recommended by the European Association of Cardiovascular Imaging and the American Society of Echocardiography.^10,11^ Among the exercise tests, the semi-supine bicycle was the most common (*n* = 2826, 71.9%), followed by the upright bicycle (*n* = 591, 15.0%) and treadmill (*n* = 515, 13.1%). Left ventricular contractile reserve (LVCR) based on the force was calculated as the ratio between stress/rest force.^11^ Left ventricular contractile reserve based on EF calculated as the stress-rest difference in EF values.^10,11^ Heart rate reserve (HRR) was calculated as stress/rest heart rate.^11^

Force represents a load-dependent index derived from SBP (numerator) and LV ESV (denominator) rather than a direct measure of myocardial contractility.

Blood pressure was a single reading measured manually (auscultatory) or automatically (oscillometry) using a calibrated sphygmomanometer at each stage of the stress protocol. The measurement at rest was obtained at the end of the preparation phase. The SBP value used for the force calculation at peak stress was the reading obtained during the final 30 s, immediately before the acquisition of peak stress images. This timing ensures that the SBP measurement corresponds as closely as possible to the haemodynamic state captured in the echocardiographic images.^10,11^

For this analysis, the HP was defined as the highest quintile of myocardial force within the study cohort. Specifically, participants with force values exceeding the 80th percentile of the study population were classified as HP. This cut-off was not pre-specified based on external guidelines, as a universal definition for myocardial hypercontractility in this context is lacking. Instead, it was empirically derived from the distribution of the data to identify the top 20% of the force, ensuring a sufficient sample size for comparative analysis against the lower quintiles. This approach allows for the investigation of physiological extremes within a representative sample of the population under study.

All centres passed the preliminary quality control for regional wall motion and LV volumes assessment as previously described.^12,13^

The same readers (one from each centre) accredited for RWMA also underwent quality control for ESV assessment. The quality control of ESV implied reading of a different set of 20 videoclips selected from seven different laboratories. The accepted threshold was ≥90% concordance with area measurement (from apical four- and two-chamber views). For each clip, the measurement was considered concordant when the planimetric area value was ±20% from the gold standard, represented by the average reading of two experienced observers of the coordinating centres.^12,13^

The index of ventricular force, also called elastance, was calculated as Force = SBP/ESV. The LVCR was calculated as the stress/rest ratio of force.^12,13^

Stress echocardiography was performed as part of a clinically driven evaluation, following the referring physician’s indications. The choice of a specific test was based on patient characteristics and the physician's clinical judgment. Typically, patients capable of exercising underwent an exercise test, while those unable to exercise received a pharmacological test. In the present analysis, patients who underwent a vasodilator stress test were excluded, as the values of LV contractile and chronotropic reserves differ from those associated with exercise or dobutamine stress.

### Data storage and analysis

The results for each test were entered in the data bank at the time of testing by each recruiting centre and sent monthly to the core lab with the electronic case report form with clinical data. After checking for internal consistency by trained technical staff, and double-checking with the centre for data verification on possibly inconsistent input, the data were added to the data bank and locked.

### Outcome data analysis

Deaths were identified from the health service database, either national or local. Non-deceased participants were contacted directly. To avoid misclassification of the cause of death, overall death was considered. Assessors were blinded to clinical, TTE, and SE results. Data were analysed by statisticians who had no role in data acquisition.

### Statistical analysis

To achieve 90% power for detecting a hazard ratio of 1.5, we required a sample size of 7500 patients. This calculation assumed a 2-year follow-up, a 2.5% annual mortality rate in the control group, and a 20% prevalence of the prognostic factor. During analysis, continuous variables were summarized as median and interquartile ranges or mean ± standard deviation according to the data distribution assessed with the D’Agostino-Pearson test. Categorical data are expressed as a number of subjects and percentages. We divided patients into Q, based on rest force results, with HP identified *a priori* as the highest Q (Q5). For continuous and categorical variables among-group differences were tested by Kruskal–Wallis and χ^2^ tests, as appropriate. The effect of force Q on survival was demonstrated by the Kaplan–Meier survival time curve. Survival curves were compared by means of the log-rank test.

In the multivariable regression, all variables that were significant in the univariable analysis (*P* < .05) were included. To avoid collinearity, parameters used in the calculation of force were not included in the final multivariable model. Furthermore, end-diastolic volume was excluded due to collinearity with ejection fraction. Among all variables tested in *Table 3*, only a small proportion (<5%) had missing values, which were imputed using the corresponding mean value.

The discriminatory performance of each model was assessed using the receiver operating characteristic (ROC) curve and its associated area under the curve, also known as the C-statistic. A formal comparison of the AUCs was performed to test for a statistically significant improvement in discrimination with the addition of new variables (e.g. Model 1 vs Model 2).

To address the potential heterogeneity introduced by the use of two different stress modalities (exercise vs dobutamine), we introduced an interaction term into the full multivariable model. We preliminary created a binary variable for ‘stress modality’ (coded as 0 for exercise and 1 for dobutamine). Subsequently, we included the interaction term between stress modality and force into a model including stress modality and force as separate variables as well as other potential confounders.

To assess whether the prognostic value of the HP is independent of the confounding effects of pre-existing left ventricular systolic dysfunction, we performed a pre-specified sensitivity analysis. By restricting the cohort to patients with preserved ejection fraction (EF ≥ 50%), we aimed to eliminate this major confounder.

Statistical significance was set at *P* < .05. All analyses were performed using R software (version 4.2.0, The R Foundation for Statistical Computing, 2022) and SPSS version 28.0.1.1.

## Results

The main demographic and clinical characteristics of patients (*n* = 10 677; age 65 ± 11 years; 6471 males, 60.6%) are given in *Table 1*. Patients with HP were older, more frequently women, with a greater prevalence of systemic hypertension.

**Table 1:** Comparison of clinical characteristics according to quintiles of force at rest

Parameters	Overall	Q1 ≤2.62 mmHg/ml	Q2 2.63–3.52 mmHg/ml	Q3 3.53–4.51 mmHg/ml	Q4 4.52–6.11 mmHg/ml	Q5 >6.11 mmHg/ml	*P* value
Number	10 677	2130	2134	2163	2161	2089	
Age (years)	65 ± 11	64 ± 12*,**	64 ± 12*,**	65 ± 11*,**	66 ± 11*	68 ± 10	<.001
Male, *n* (%)	6471 (60.6%)	1661 (78.0%)*,**,***,****	1475 (69.1%)*,**,***	1363 (63%)*,**	1109 (51.3%)*	863 (41.3%)	<.001
BMI (kg/m^2^)	28.2. ± 6.7	29.5 ± 9.8*,***,****	29.0 ± 7.3*,***	27.9 ± 4.5	27.2 ± 4.1	26.7 ± 4.4	<.001
BSA (m^2^)	1.89 ± 0.21	2.0 ± 0.22*,**,***,****	1.94 ± 0.22*,**,***	1.87 ± 0.18*,**	1.81 ± 0.18*	1.74 ± 0.18	<.001
LBBB, *n* (%)	704 (6.8%)	280 (13.5%)*,**,***,****	168 (8.0%)*,**,***	104 (4.9%)*	87 (4.2%)	65 (3.3%)	<.001
History of AF, *N* = 3781 (%)	434 (11.5%)	152 (17.9%)*,**,***,****	106 (11.7%)	87 (9.6%)	59 (8.1%)	30 (7.6%)	<.001
Hypertension (*n*, %)	8075 (75.6%)	1592 (74.7%)*	1605 (75.2%)*	1598 (73.9%)*	1626 (75.2%)*	1654 (79.2%)	.001
Diabetes, *n* (%)	3114 (29.2%)	669 (31.4%)*,**,***,****	564 (26.4%)*	540 (25.0%)*	570 (26.4%)*	771 (36.9%)	<.001
NYHA class (*N* = 2687)		*,**,***,****					<.001
NYHA Ⅰ, *n* (%)	1919 (71.4%)	273 (56.6%)	467 (74.1%)	532 (75.6%)	417 (73.8%)	230 (75.2%)	
NYHA Ⅱ, *n* (%)	643 (23.9%)	149 (30.9%)	141 (22.4%)	154 (21.9%)	130 (23.0%)	69 (22.5%)	
NYHA Ⅲ, *n* (%)	125 (4.7%)	60 (12.4%)	22 (3.5%)	18 (2.6%)	18 (3.2%)	7 (2.3%)	
β-blocker, *n* (%)	5373 (49.5%)	1228 (57.7%)*,**,***,****	1093 (51.2%)**	1082 (50.0%)**	956 (44.3%)	1014 (48.5%)	<.001
ACE inhibitors or ARBs (*N* = 3834), *n* (%)	2496 (65.1%)	528 (61.5%)	605 (65.5%)	612 (66.9%)	491 (66.4%)	260 (65.5%)	.142
Calcium channel blockers (*N* = 3834), *n* (%)	1068 (27.9%)	243 (28.3%)	279 (30.2%)	232 (25.4%)	200 (27.0%)	114 (28.7%)	.206
Diuretics (*N* = 3834), *n* (%)	1043 (27.2%)	325 (37.8%)*,**,***,****	257 (27.8%)	202 (22.1%)	175 (23.6%)	84 (21.2%)	<.001
Prior myocardial infarction, *n* (%)	2682 (25.1%)	827 (38.8%)*,**,***,****	572 (26.8%)*,**,***	522 (24.1%)*,**	374 (17.3%)	387 (18.5%)	<.001
Prior coronary revascularization, *n* (%)	3658 (34.3%)	976 (45.8%)*,**,***,****	779 (36.5%)*,**,***	705 (32.6%)**	587 (27.2%)	611 (29.2%)	<.001

^*^ *P* < .05 vs Q5.

^**^ *P* < .05 vs Q4.

^***^ *P* < .05 vs Q3.

^****^ *P* < .05 vs Q2.

### Resting transthoracic echocardiography

On average, EF was 59.3 ± 8.8% and force was 4.52 ± 2.11 mmHg/ml (Q3 = 3.50–4.26 mmHg/ml). Patients in the HP group demonstrated higher values of EF and lower values of LVEDV, LVESV, and SV (*Table 2*).

**Table 2:** Comparison of resting TTE findings according to quintiles of force at rest

	Overall	Q1 ≤2.62 mmHg/ml	Q2 2.63–3.52 mmHg/ml	Q3 3.53–4.51 mmHg/ml	Q4 4.52–6.11 mmHg/ml	Q5 >6.11 mmHg/ml	*P* value
Number	10 677	2130	2134	2163	2161	2089	
EDV (ml)	91.2 ± 38.6	144.1 ± 41.1*,**,***,****	102.3 ± 19.3*,**,***	86.6 ± 15.1*,**	72.0 ± 13.9*	52.1 ± 14.9	<.001
ESV (ml)	38.3 ± 23.5	72.2 ± 29.8*,**,***,****	42.6 ± 7.0*,**,***	33.0 ± 4.7*,**	26.2 ± 3.9*	17.3 ± 4.1	<.001
EF (%)	59.3 ± 8.8	51.0 ± 10.2*,**,***,****	57.8 ± 6.6*,**,***	60.7 ± 6.12*,**	62.7 ± 6.4*	64.8 ± 6.9	<.001
WMSI	1.11 ± 0.26	1.31 ± 0.43*,**,***,****	1.09 ± 0.22*,**,***	1.06 ± 0.17*,**	1.04 ± 0.14	1.03 ± 0.11	<.001
SV (ml)	53.0 ± 20.1	72.0 ± 23.4*,**,***,****	59.8 ± 15.4*,**,***	52.6 ± 13.1*,**	45.8 ± 12.2*	34.8 ± 12.4	<.001
B-lines *n* = 8057	1248 (15.5%)	338 (21.7%)*,**,***,****	234 (13.5%)	250 (13.8%)	252 (14.1%)	174 (14.8%)	<.001
LAVI (ml/m^2^), *n* = 3218	24.4 ± 11.7	33.1 ± 13.8*,**	28.0 ± 12.4*,**,***	24.6 ± 10.5	24.6 ± 10.0	24.4 ± 11.7	<.001
LVMI (mg/m^2^) *n* = 2471	90.1 ± 28.5	103.1 ± 31.1*,**,***,****	91.0 ± 27.3*,**,***	84.9 ± 24.5	82.9 ± 24.8	77.6 ± 21.0	<.001
E/e’ *N* = 1446	9.0 ± 4.2	9.9 ± 5.4***,****	8.6 ± 3.4	8.4 ± 3.4	9.2 ± 4.5	9.1 ± 3.5	<.001
sPAP (mmHg) *N* = 1407	30.0 ± 20.5	32.3 ± 18.4*,**	29.8 ± 16.9	30.9 ± 18.6	27.3 ± 8.5	27.8 ± 8.6	.014
GLS (%) *N* = 1318	−17.2 ± 3.4	−14.0 ± 4.3*,**,***,****	−17.1 ± 3.2*,**,***	−18.2 ± 3.0****	−18.5 ± 3.1	−18.8 ± 4.1	<.001

LAVI, left atrial volume index; EDV, left ventricular end-diastolic volume; ESV, left ventricular end-systolic volume; EF, left ventricular ejection fraction; LVMI, left ventricular mass index; SV, stroke volume; WMSI, wall motion score index.

^*^ *P* < .05 vs Q5.

^**^ *P* < .05 vs Q4.

^***^ *P* < .05 vs Q3.

^****^ *P* < .05 vs Q2.

### Outcome prediction

During a median of 24 months (interquartile range = 13–40 months) of follow-up, 509 deaths occurred.

**Figure 2:**
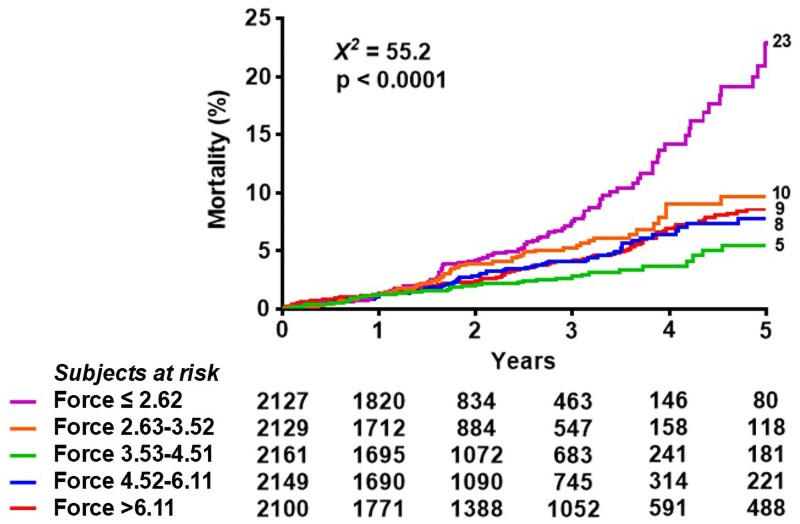
Kaplan–Meier curve for all-cause death in the 5 Qs

Patients were stratified into quintiles of contractile force (systolic pressure/end-systolic volume). Mortality rates followed a *U*-shaped pattern across the spectrum (*Figure 2*). Q1, representing the lowest force values (≤2.62 mmHg/mL), showed the highest mortality (31.8%), consistent with advanced systolic dysfunction. Survival improved progressively through Q2–Q3, reaching its optimum in Q3 (13.4%), which likely represents physiological contractility. Beyond this range, mortality increased again in Q5—the hypercontractile phenotype (HP, >6.11 mmHg/mL)—with a 24.6% death rate. The difference in survival between quintiles was statistically significant (log-rank *P* < .001), confirming that both reduced and excessive contractile force are associated with adverse prognosis.

**Figure 3:**
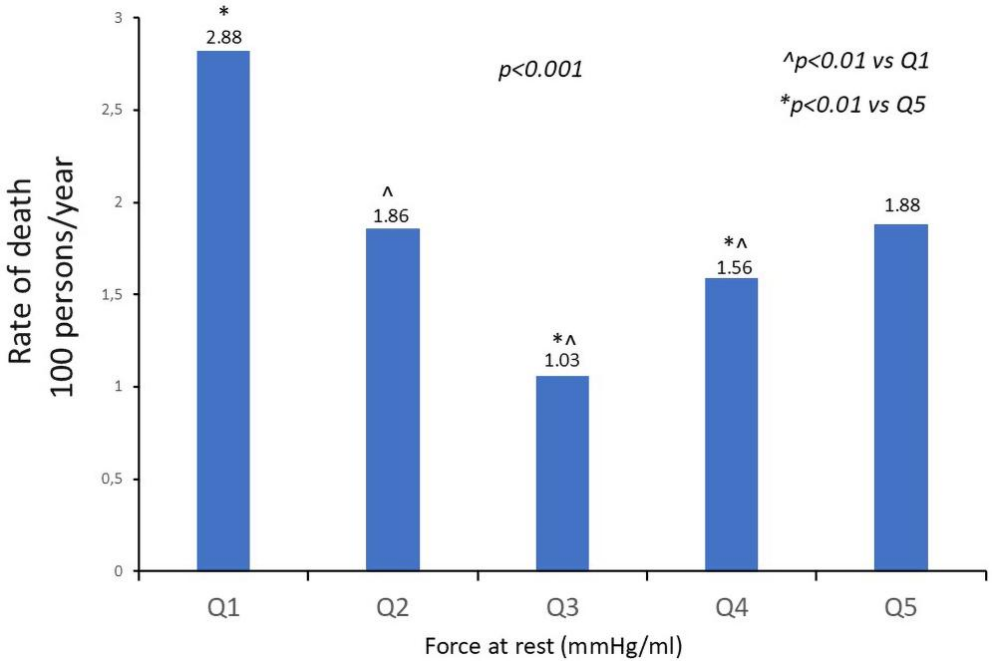
Annual mortality according to the resting force values in the Q5. *, *P* < .001 vs Q5 and ^ *P* < .001 vs Q1

Similarly, the annualized mortality rate was lowest for Q3 (1.03 events per 100 person-years), and highest for Q1 (2.88 events per 100 person-years), intermediate for Q5 (1.88 events per 100 person-years), Q2 (1.86 events per 100 person-years), and Q4 (1.56 events per 100 person-years) (*Figure 3*).

The multivariable Cox regression analysis identified LVEF and force Q1, Q2, and Q5 as independent predictors of mortality (see *Table 3*).

**Table 3:** Univariable and multivariable predictors of all-cause death in Cox analysis with resting TTE variables in 10 677 patients

	Univariable Cox Regression analysis		Multivariable Cox Regression analysis	
Variables	Hazard ratio (95% CI)	*P*	Hazard ratio (95% CI)	*P* value
Age (years)	1.065 (1.055–1.075)	<.001	1.062 (1.052–1.073)	**<**.**001**
Sex (male)	1.292 (1.073–1.555)	.007	1.274 (1.050–1.547)	.**014**
Hypertension	1.201 (0.970–1.487)	.093		
Beta-blocker therapy	1.123 (0.918–1.374)	.151		
Diabetes mellitus	2291 (1.913–2.774)	<.001	1.819 (1.514–2.186)	**<**.**001**
Prior MI	1.573 (1.310–1.888)	<.001	1.128 (0.932–1.363)	.215
Rest HR (b/m)	1.006 (0.999–1.014)	.110		
Rest SBP (mmHg)^a^	1.001 (0.996–1.006)	.601		
Rest EDV (ml)^a^	1.005 (1.004–1.007)	<.001		
Rest ESV (ml)^a^	1.010 (1.008–1.010)	<.001		
Rest EF (%)	0957 (0.939–0.975)	<.001		
Ejection fraction at rest (%)	0952 (0.945–0.959)	<.001	0.963 (0.953–0.972)	**<**.**001**
Force at rest (Q3) (mmHg/ml)				
Q1	2.847 (2.081–3.986)	<.001	1.686 (1.197–2.375)	.**003**
Q2	1.884 (1.350–2.628)	.001	1.635 (1.168–2.288)	.**004**
Q4	1.401 (1.003–1.956)	.048	1.345 (0.961–1.882)	.084
Q5	1.540 (1.130–2.097)	.006	1.531 (1.116–2.099)	.**008**

HR, heart rate; SBP, systolic blood pressure; EDV, left ventricular end-diastolic volume; ESV, left ventricular end-systolic volume; EF, left ventricular ejection fraction.

^a^Variables were excluded from the multivariable model for the presence of collinearity.

Additionally, we forced into the multivariable regression model all variables that showed a significant difference across force quintiles (*Table 1*), were not collinear, and had no missing data: body mass index, left bundle branch block, and prior coronary revascularization. The inclusion of these variables did not change the results of the multivariable logistic regression model or the role of force quintiles as predictors of all-cause mortality, as reported in *Table 3*. We added a Force × EF interaction term to the primary Cox model. The interaction was not statistically significant in univariate analysis (*P* = .840, HR 1.00, 95% CI: 0.99–1.00), suggesting that the prognostic effect of force does not vary significantly across the whole range of EF in our preserved EF cohort.

**Figure 4:**
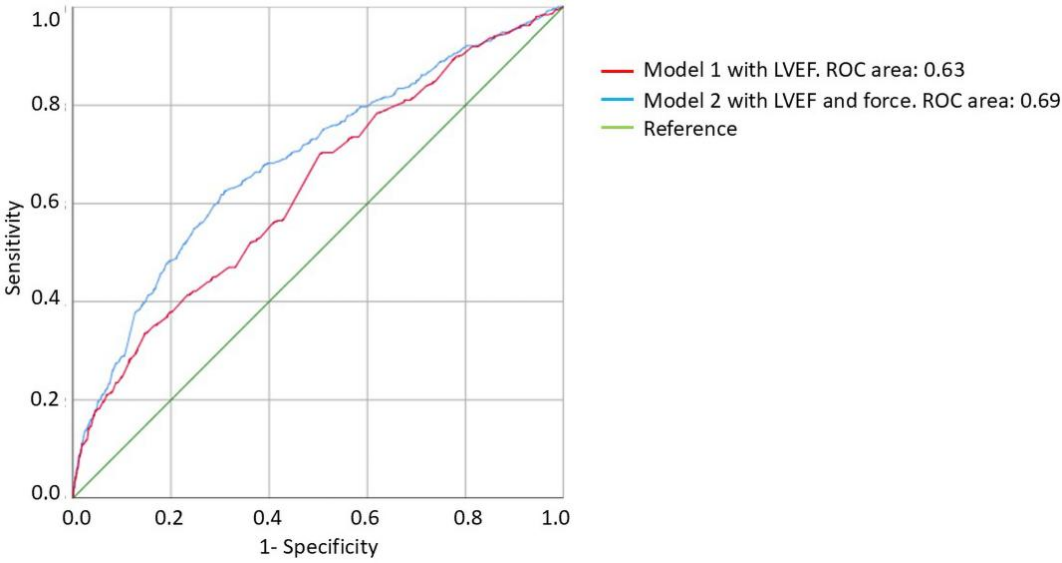
Prediction of the probability of death with all-cause death prediction model. ROC curve analysis with LVEF (model 1, red line) and with LVEF and force (model 2, blue line)

For the endpoint of mortality, the discriminatory performance of LVEF (Model 1) evaluated with ROC curve analysis was improved with the addition of Force (Model 2, area under ROC curve = 0.69, 95% confidence interval: 0.66–0.71 vs Model 1, area under ROC curve = 0.63, 95% confidence interval: 0.60–0.66, D + 9%, *P* < .001, *Figure 4*).

We conducted a subgroup analysis restricted to 9526 patients with baseline LVEF ≥50% (Supplementary Table S1).

The values of quintiles were obviously different in this population, with Q5 corresponding to values >6.32 mmHg/ml, but still, at multivariable analysis, Q5 remained a significant predictor of reduced survival (Supplementary Table S2).

Mortality rate was higher in Q5 compared to the other groups (Supplementary Figure S1).

### Stress echocardiography

We studied 5834 patients with exercise (3932, 67%) or dobutamine (1902, 33%). The abnormal force-based LVCR was defined ≤1.6, according to the ROC curve analysis (sensitivity: 55.7%, specificity: 58.1%, area under ROC curve: 0.575, 95% confidence interval: 0.562–5.587). Patients with HP (Q5, 707, 12%) showed reduced LVCR based on force or EF (ΔEF: Q5 = 4.3 ± 9.4% vs Q1–Q4 = 7.0 ± 9.3%; *P* < .001; Δ-Force: Q5 = 1.79 ± 0.33 vs Q1–Q4 = 1.90 ± 0.83, *P* < .001) and blunted HRR (Q5 = 1.79 ± 0.33 vs Q1–Q4 = 1.85 ± 0.38; *P* < .01) (*Table 4*).

**Table 4:** Comparison of stress echocardiography findings according to quintiles of force at rest

	Overall	Q1 ≤2.62 mHg/ml	Q2 2.63–3.52 mmHg/ml	Q3 3.53–4.51 mmHg/ml	Q4 4.52–6.11 mmHg/ml	Q5 >6.11 mmHg/ml	*P* value
Number	*n* = 5834	1162	1563	1343	1059	707	
WMSI	1.17 ± 0.33	1.33 ± 0.46**	1.14 ± 0.27**	1.12 ± 0.26**	1.11 ± 0.25**	1.09 ± 0.20	<.001
ΔWMSI	0.062 ± 0.225	0.065 ± 0.276	0.058 ± 0.208	0.061 ± 0.203	0.074 ± 0.022	0.049 ± 0.172	.203
Stress-induced ischaemia	1258 (21.6%)	417 (26.7%)**	282 (21.0%)*	241 (20.7%)	209 (19.7%)	109 (15.4%)	<.001
B-lines *n* = 1752	552 (29.8%)	144 (34.3%)	107 (25.3%)	105 (27.7%)	97 (29.4%)	69 (34.5%)	.026
Δ-EF	6.7 ± 9.3	6.7 ± 9.4**	7.6 ± 9.1**	7.1 ± 9.2**	6.3 ± 9.2**	4.3 ± 9.4	<.001
Δ-Force	1.88 ± 0.82	1.87 ± 0.88**	1.91 ± 0.85**	1.93 ± 0.81**	1.89 ± 0.77**	1.72 ± 0.70	<.001
Abnormal Δ-Force	2472 (42.4%)	689 (44.1%)*	548 (40.8%)**	447 (38.5%)**	426 (40.2%)**	362 (51.2%)	<.001
HRR	1.85 ± 0.38	1.89 ± 0.41**	1.88 ± 0.41**	1.84 ± 0.96	1.79 ± 0.95	1.79 ± 0.33	<.001

EF, ejection fraction; HRR, heart rate reserve; WMSI, wall motion score index.

^*^ *P* < .0.05 (Q5 vs all other groups).

^**^ *P* < .001 (Q5 vs all other groups).

When only patients with normal EF (≥50%) were considered, there was a higher prevalence of stress-induced B-lines in HP group (Q5 = 69/200, 34.5% vs Q1–Q4 = 354/1335, 26.5%, *P* = .013) and reduced LVCR (ΔEF: Q5 = 4.2 ± 9.0% vs Q1–Q4 = 6.8 ± 98.7%; *P* < .001; Δ-Force: Q5 = 1.72 ± 0.70 vs Q1–Q4 = 1.93 ± 0.84, *P* < .001).

In the 5834 patients with SE, follow-up duration was 20 months (interquartile range = 12–30 months) and 185 deaths occurred.

**Figure 5:**
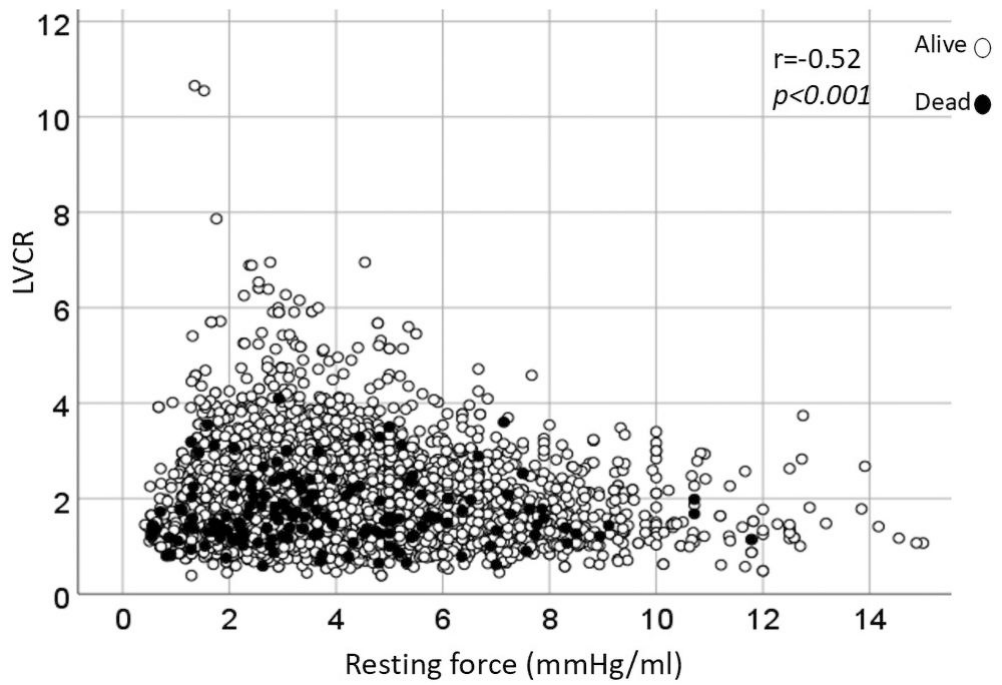
Correlation between resting force (x-axis) and LVCR (y-axis). Black dots indicate dead patients, and white dots those alive at follow-up

In a Cox model including variables of the basic model as well as LVEF, force, and stress variables such as force-based LVCR (*Table 5*), stress-rest change in EF [hazard ratio, (HR): 0.981, 95% coefficient interval (CI): 0.970–0.993, *P* = .001], Q5 of force at rest (HR: 1.589, 95% CI: 1.141 2.211, *P* = .006), and abnormal force-based LVCR ≤1.60 (HR: 1.654, 95% CI: 1.344–2.036, *P* < .001) were significant predictors of death (*Table 5*). All patients with LV contractile reserve >4.1 (present in 185 patients, 3.2% of the population) survived (*Figure 5*).

**Table 5:** Univariable and multivariable predictors of all-cause death in Cox analysis with resting TTE and SE variables in 5834 patients

	Univariable Cox Regression analysis		Multivariable Cox Regression analysis	
Variables	Hazard ratio (95% CI)	*P*	Hazard ratio (95% CI)	*P* value
Age (per year)	1.061 (1.045–1.077)	<.001	1.062 (1.051–1072)	<.001
Sex (male)	1.849 (1.341–2.548)	<.001	1.314 (1.073–1607)	.008
Hypertension	1003 (0.726–1.386)	.983		
Beta-blocker therapy	1.041 (0.740–1.465)	.818		
Diabetes mellitus	1.855 (1.379–2.494)	<.001	1.574 (1.299–1.908)	<.001
Prior MI	2.291 (1.711–3.068)	<.001	1.039 (0.850–1.1270)	.709
Rest HR (b/min)	1.008 (0.997–1.019)	.170		
Rest SBP (mmHg)^a^	1.008 (1.000–1.016)	.039		
Rest EDV (ml)^a^	1.006 (1.002–1.009)	<.001		
Rest ESV (ml)^a^	1.010 (1.006–1.014)	<.001		
Ejection fraction at rest (%)^a^	0.941 (0.915–0.969)	<.001		
Force at rest (Q3) mmHg/ml				
Q1	2.260 (1.363–3.746)	.002	1.539 (1.069–2.217)	.020
Q2	1.846 (1.081–3.152)	.009	1.605 (1.133–2.274)	.008
Q4	1.910 (1.105–3.301)	.020	1.316 (0.927–1.868)	.125
Q5	1.975 (1.103–3.533)	.022	1.589 (1.141–2.211)	.006
LV CR: Abnormal Δ Force (≤1.6)	2.010 (1.956–2.838)	<.001	1.654 (1.344–2.036)	<.001
Δ EF (%)	0.971 (0.962–0.981)	<.001	0.981 (0.970–0993)	.001

HR, heart rate; SBP, systolic blood pressure; EDV, left ventricular end-diastolic volume; ESV, left ventricular end-systolic volume; EF, left ventricular ejection fraction; CR, contractile reserve.

^a^Variables were excluded from the multivariable model for the presence of collinearity.

### Interaction analyses by stress modality

There was no significant effect (*P* = .10) in the interaction analysis of exercise versus dobutamine on the relationship between resting force and mortality in the multivariable model adjusted for age, sex, diabetes, previous myocardial infarction, and resting ejection fraction.

## Discussion

In this study, we used rest TTE with force to characterize distinct LV contractile phenotypes at rest. The main findings can be summarized as follows: (1) patients with HP were more often older, female, with a higher prevalence of hypertension or diabetes mellitus, and with a lower prevalence of left bundle branch block, previous myocardial infarction, or prior coronary revascularization. (2) At rest, they exhibited smaller LV volumes and higher EF values. (3) Despite supernormal resting contractility, their hearts were functionally more vulnerable, with reduced contractile and chronotropic reserve, and greater pulmonary congestion despite less ischaemia. (4) They had worse survival, with increased force conferring prognostic value independent of the underlying LV EF phenotype and age. (5) A supernormal force-based LVCR was associated with an excellent prognosis (*Graphical Abstract*).

### Mechanistic determinants of hypercontractile phenotype

The HP of the LV may arise from three principal mechanisms: intrinsic myosin overactivity (myocardial), increased central arterial stiffness (aortic), and heightened sympathetic drive (neurogenic). Experimental models show that excessive LV contractility, driven by enhanced myosin activity, creates an energy-demanding state that predisposes to maladaptive remodelling and long-term dysfunction.^14^ By analogy, the HP in CCS may represent an early stage of latent LV dysfunction due to the chronically increased myocardial oxygen demand. Consistently, patients with this phenotype demonstrated reduced contractile and chronotropic reserve.

The paradox wherein supernormal organ function at rest signifies a poor prognosis, while a supernormal functional reserve during stress indicates a protective prognosis, is a recognized physiological concept beyond cardiac contractility. The presented evidence hits upon a fundamental principle in physiology and systems medicine, often discussed in the context of allostasis and allostatic load. Allostasis is the process of achieving stability (or ‘homeostasis’) through change. This is the *normal, adaptive* stress response (e.g. a rise in cardiac output during exercise). This is the ‘supernormal function during stress’ associated with a good prognosis. Allostatic load is the ‘wear and tear’ on the body and brain resulting from chronic overactivity or underactivity of allostatic systems. This is the *maladaptive, long-term cost* of forced adaptation.^15^

### Comparison with previous studies

Our findings are broadly consistent with previous evidence showing that a supernormal EF is prognostically unfavourable in patients with CCS or heart failure with preserved EF.^16–22^ Fewer data are available regarding LV force, which has generally shown stronger prognostic relevance than EF in patients with depressed or normal EF.^23–25^ Reported thresholds for identifying HP and increased prognostic vulnerability appear to be disease-specific, with values >5.48 mmHg/mL in heart failure with preserved EF^5^ and >7.32 mmHg/mL in hypertrophic cardiomyopathy with preserved EF, where LV outflow tract gradient is added to systolic blood pressure (SBP) in the force calculation.^6^ In line with these reports, our study demonstrated a 70% increase in mortality among CCS patients with resting LV force >6.11 mmHg/mL.

Previous work also showed that HP is associated with echocardiographic features suggestive of impaired diastolic function, such as small LV chamber size.^4,5^ The present study confirms these observations and extends current knowledge by assessing prognostic value in an unselected CCS population referred for SE testing, considering all-cause mortality as the sole endpoint within a large-scale, multicentre design. Importantly, prognostic assessment was enriched by a composite functional evaluation combining resting TTE and SE results. This approach allowed us to link anatomical features to clinical outcomes through functional parameters, highlighting the multilayered vulnerability of HP—from impaired contractile reserve to reduced chronotropic reserve (indicative of impaired sympathetic and autonomic function) and diastolic dysfunction, indicated by the increased number of B-lines during stress. The hypothesized mechanism underlying a preserved contractile reserve might be the absence of ischaemia, necrosis, or fibrosis.^10,11^

### Clinical implications

Hypercontractile phenotype is a distinct pathophysiological entity and a unique endotype within the clinical phenotype of CCS. Ejection fraction reflects predominantly LV circumferential shortening, and it can be normal or increased with a reduction of contractile longitudinal function assessed with global longitudinal strain, and should not be used as a stand-alone measure of LV function.^26^ Force integrates the role of loading in assessing LV function and has stronger theoretical and experimental foundations for identifying HP compared to EF, which is notably influenced by heart rate, loading conditions, and LV size.^27^

The ability to safely and efficiently identify a specific HP endotype using resting TTE is a critical prerequisite for targeted therapeutic interventions, such as beta-blockers or, potentially, novel cardiac myosin inhibitors.^28^ However, therapeutic implications should be proposed with caution, since, for instance, HP have small ventricular volumes and limited stroke volume reserve, suggesting a physiology that may be relatively heart-rate dependent. In such circumstances, heart-rate-reducing therapies could theoretically reduce cardiac output rather than improve it. Recommendations regarding specific pharmacologic strategies remain speculative and should be corroborated by future randomized studies driven by the non-invasive recognition of specific phenotypes.

### Study limitations

First, the dataset was derived from multiple institutions—including both academic centres and community hospitals—using different imaging vendors and stress protocols, which may introduce inter-centre variability. Nevertheless, the study benefited from broad inclusion, enrolling patients from 12 countries across Europe and the Americas, and reflecting local practices, expertise, and patient profiles. This pragmatic and adaptable approach enhances the external validity of the findings, making them more representative of real-world clinical settings. Second, although no central core-lab adjudication was performed, all participating sites contributed data after undergoing preliminary quality control, ensuring consistency and reliability of database entry.

Volumetric echocardiography traditionally relies on manual delineation of endocardial contours, a method susceptible to non-negligible variability—even in expert laboratories—despite rigorous quality control and acceptable inter-operator reproducibility for end-systolic area measurements. At present, accuracy remains dependent on image quality and correct probe alignment, particularly in suboptimal acoustic windows. However, the introduction of automated algorithms for click-free assessment of LV volumes by the integration of artificial intelligence will significantly enhance both the speed and accuracy of volumetric echocardiography. This advancement enables rapid, objective, and reproducible assessment, at rest and during stress, of a parameter that was traditionally labour-intensive, time-consuming, and operator-dependent.^29^ Other imaging invasive and non-invasive techniques may be more accurate and reproducible than TTE for assessing LV volumes, but the low cost, universal availability, radiation-free, and environment-friendly nature of ultrasound make it unmatched for a first-line application.^30^

The definition of the HP based on quintiles of force, although statistically clear, depends on the cohort distribution and oversimplifies a continuous variable, which may limit extrapolation to other populations. In fact, the cut-off values can be different in other populations, and typically higher in a population of hypertrophic cardiomyopathy^6^ and lower in a population of patients with heart failure and preserved ejection fraction.^5^

The primary endpoint of all-cause mortality does not distinguish between cardiovascular and non-cardiovascular deaths, thereby reducing clinical specificity. However, death is a binary, undeniable event. There is no interpretation, no judgment, and no bias. It's the hardest endpoint that can be verified with 100% certainty through public records.

While we adhered to a strict protocol for measuring blood pressure concurrently with image acquisition, the use of a single reading rather than an averaged value may introduce variability. In both stress modalities (exercise or dobutamine), a single reading was used for the peak stress force calculation, as capturing multiple averaged readings during the brief window of true peak stress is not feasible without delaying image acquisition.

Exercise and dobutamine SE represent fundamentally different physiological stimuli. Exercise induces a complex haemodynamic response involving increases in heart rate, preload, and metabolic demand, whereas dobutamine produces a pharmacologically mediated increase in contractility with distinct effects on loading conditions. Therefore, while acknowledging the distinct physiological stimuli, pooling the modalities in the primary analysis was justified, as formal testing for interaction did not reveal a significant effect modification by stress modality.

Q5 patients showed a lower prevalence of left bundle branch block, prior myocardial infarction, and previous coronary revascularization, suggesting that the underlying ischaemic substrate may not be comparable between groups, but the objective measures of CAD disease severity were missing in the majority of patients due to a lack of coronary angiographic information at the time of evaluation. Therefore, residual confounding by CAD burden cannot be excluded.

Our model was intentionally constructed to isolate the independent prognostic value of high force quintile after adjusting for a core set of well-established clinical and echocardiographic confounders, including demographics, diabetes, and ejection fraction. However, a more comprehensive adjustment for structural heart disease would strengthen the analysis, and the next step on an expanded series with longer follow-up is to incorporate HP with other established markers of structural and haemodynamic burden derived from rest TTE and SE, such as atrial enlargement, RWMA, LV volumes, and pulmonary congestion.

## Conclusion

In CCS, HP at rest is a maladaptive feature associated with reduced ventricular filling, impaired contractile and chronotropic reserve during stress, and increased all-cause mortality. This paradox reveals that a ‘stronger’ heart by conventional metrics is functionally and prognostically weaker.

## Supplementary Material

xvag151_Supplementary_Data

## Declarations

### Data Availability

The data that support the findings of this study are available from the corresponding author upon reasonable request.

### Pre-registered Clinical Trial Number

ClinicalTrials.govIdentifier NCT 030.49995; ClinicalTrials.govIdentifier NCT NCT050.81115
