# Supplementary material for: Hypercontractile phenotype at rest in chronic coronary syndromes predicts impaired functional reserve and increased mortality
Source: ESC Heart Fail. 2026 Jun 6;13(4):xvag151. doi: 10.1093/eschf/xvag151 (PMC13344841; doi:10.1093/eschf/xvag151)
Supplement: xvag151_Supplementary_Data [file xvag151_supplementary_data.zip › suppl table.docx]

**Table S1** Comparison of clinical characteristics according to quintiles of force at rest

| Parameters | Overall | Q1  ≤2.90  mmHg/ml | Q2  2.91-3.74  mmHg/ml | Q3  3.75-4.68  mmHg/ml | Q4  4.69-6.32 mmHg/ml | Q5  >6.32 mmHg/ml | *P value* |
| --- | --- | --- | --- | --- | --- | --- | --- |
| Number | **9526** | **1941** | **1907** | **1860** | **1916** | **1902** |  |
| Age (yrs) | 65 ±11 | 63 ±12*^ | 64±12*^ | 64±11*^ | 65±10* | 68±10 | <0.001 |
| Male, n (%) | 5600 (58.8%) | 1412 (74.0%)*^§$ | 1283 (70.5%)*^§ | 1162 (59.9%)*^ | 963 (50.3%)* | 780 (41.0%) | <0.001 |
| BMI (kg/m^2^) | 28.2.±6.7 | 29.5±9.8*§$ | 29.0±7.3*§ | 27.9±4.5 | 27.2±4.1 | 26.7±4.4 | <0.001 |
| BSA (m^2^) | 1.89±0.21 | 2.05±0.23*^§$ | 1.97±0.21*^§ | 1.89±0.18*^ | 1.83±0.18* | 1.75±0.18 | <0.001 |
| History of AF,  N=3379 (%) | 373 (11.0%) | 132 (16.8%)*^§$ | 90 (11.1%) | 71 (8.8%) | 53 (8.3%) | 27 (8.0%) | <0.001 |
| Hypertension (n, %) | 7210 (75.7%) | 1396  (73.2%)* | 1415  (76.1%)* | 1426  (73.1%)* | 1465 (76.5%)* | 1509 (79.3%) | 0.001 |
| Diabetes, n (%) | 2675 (28.1%) | 534 (28.0%)*^§$ | 479  (25.8%)* | 462 (23.9%)* | 486 (25.4%)* | 713 (37.5%) | <0.001 |
| 𝛽-blocker, n (%) | 4651 (48.4%) | 983 (51.6%)*^§$ | 944 (50.8%)^ | 942 (48.5%)^ | 861 (45.0%) | 921 (48.4%) | <0.001 |
| Prior myocardial infarction, n (%) | 2090 (21.9%) | 520 (27.3%)*^§$ | 482 (25.9%)*^§ | 421 (21.7%)*^ | 3.3 (18.1%) | 344 (16.5%) | <0.001 |
| Prior coronary revascularization, n (%) | 3098 (32.5%) | 736 (38.6%)*^§$ | 665 (35.8%)*^§ | 617 (31.2%)^ | 522 (27.2%) | 558 (29.3%) | <0.001 |

*p<0.05 vs Q5

^p<0.05 vs Q4

§p<0.05 vs Q3

$p<0.05 vs Q2

**Table S2: Univariable and multivariable predictors of all-cause death in Cox analysis with resting TTE variables in 9,526 patients with LVEF≥50%**

|  | **Univariable Cox**  **Regression Analysis** | | **Multivariable Cox**  **Regression Analysis** | |
| --- | --- | --- | --- | --- |
| **Variables** | **Hazard ratio (95%CI)** | **P** | **Hazard ratio (95%CI)** | **P** |
| **Age (years)** | 1.068 (1.056-1.080) | <.001 | 1.067 (1.055-1.079) | <.001 |
| **Sex (male)** | 1.247 (1.010-1.539) | .040 | 1.341 (1.055-1.079) | .009 |
| **Hypertension** | 1.366 (1.057-1.765) | .017 | 1.029 (0.993-1.336) | 0.828 |
| **Beta-blocker therapy** | 1.123 (0.918-1.374) | .259 |  |  |
| **Diabetes mellitus** | 2.320 (1.884-2.858) | <.001 | 1.911 (1.544-2.366) | <.001 |
| **Prior MI** | 1.316 (1.052-1.647) | .016 | 1.098 (0.869-1.376) | .446 |
| **Rest HR (b/m)** | 1.003 (0.995-1.012) | .438 |  |  |
| **Rest SBP (mmHg)*** | 1.001 (1.001-1.013) | .015 |  |  |
| **Rest EDV (ml)*** | 1.002 (0.999-1.005) | 0.151 |  |  |
| **Rest ESV (ml)*** | 1.007 (1.001-1.013) | .015 |  |  |
| **Ejection fraction at rest (%)** | 0.957 (0.939-0.975) | <.001 | 0.971 (0.951-0.991) | .006 |
| **Force at rest (Q3) (mmHg/ml)** |  |  |  |  |
| **Q1** | 2.175 (1.472-3.213) | <.001 | 1.942 (1.302-2.895) | .001 |
| **Q2** | 1.778 (1.196-2.643) | .004 | 1.708 (1.146-2.545) | .009 |
| **Q4** | 1.690 (1.154-2.476) | .007 | 1.507 (1.238-2.549) | .037 |
| **Q5** | 1.969 (1.1.385-2.801) | <.001 | 1.776 (1.238-2.549) | <.001 |

***** Variables were excluded from the multivariable model for the presence of collinearity

HR: heart rate, SBP: systolic blood pressure, EDV, left ventricular end-diastolic volume; ESV, left ventricular end-systolic volume; EF, left ventricular ejection fraction
