# Supplementary figures and images for: Hypercontractile phenotype at rest in chronic coronary syndromes predicts impaired functional reserve and increased mortality
Source: ESC Heart Fail. 2026 Jun 6;13(4):xvag151. doi: 10.1093/eschf/xvag151 (PMC13344841; doi:10.1093/eschf/xvag151)

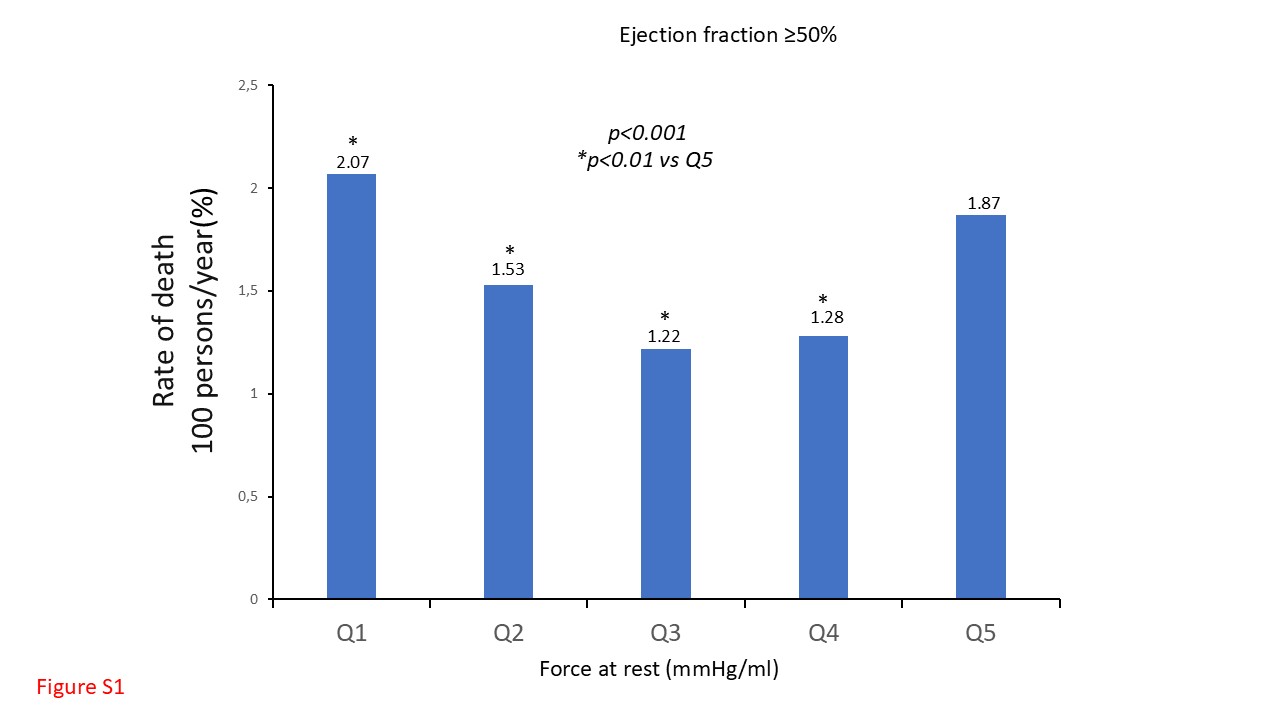

Supplement: xvag151_Supplementary_Data [file xvag151_supplementary_data.zip › Figure S1.JPG]
